# Diferences in concetration of transforming growth factor beta and interferon gama in adults with major depressive disorder regarding on seveirty of disorder

**DOI:** 10.1192/j.eurpsy.2025.1349

**Published:** 2025-08-26

**Authors:** A. M. Munjiza Jovanovic, M. Kostic, D. Pesic, M. Jeremic, I. Markovic

**Affiliations:** 1 Institute of Mental Health; 2 Faculty of Medicine, University of Belgrade; 3 Institute for Biochemistry, Belgrade, Serbia

## Abstract

**Introduction:**

Over the past 30 years, cytokines have been extensively studied in relation to neuroplasticity, modulation of neuro-signaling, and various psycho-immunological aspects of depression. Interferon gamma (INF-γ) is traditionally recognized for its pro-inflammatory role, but it also possesses regulatory functions by antagonizing Transforming Growth Factor beta (TGF-β). TGF-β acts as an anti-inflammatory and regulatory cytokine. The interaction between these cytokines remains inadequately explored in patients with depression.

**Objectives:**

The aim of our study was to analyze the differences in serum concentrations of INF-γ and TGF-β between patients with Major Depressive Disorder (MDD) and healthy controls.

**Methods:**

Blood samples were obtained from 55 patients who met the DSM-IV-R criteria for a current MDD episode without psychotic symptoms, and from 45 healthy controls, matched for age and gender. Participants were assessed using the Beck Depression Inventory (BDI) and the Hamilton Depression Rating Scale (HDRS). Serum concentrations of INF-γ and TGF-β were determined using enzyme-linked immunosorbent assays.

**Results:**

The concentration of TGF-β was significantly higher in patients compared to healthy controls, while no differences were observed for INF-γ between the groups. Moreover, INF-γ concentrations were statistically higher in patients with more severe depression, as measured by HDRS and BDI. TGF-β levels increased with severity only as assessed by HDRS. Among various factors (sociodemographic, clinical, and hereditary), INF-γ was positively correlated solely with the number of hospitalizations, whereas TGF-β levels correlated with the duration of treatment and symptoms.

**Conclusions:**

The interplay between TGF-β and INF-γ is frequently considered a crucial component of the inflammatory hypothesis in depression. Further research is needed to understand why concentrations are elevated in patients with more severe forms of depression, yet show no correlation with other clinical or sociodemographic factors.

**Disclosure of Interest:**

None Declared

